# Divergent Capacity of Scleractinian and Soft Corals to Assimilate and Transfer Diazotrophically Derived Nitrogen to the Reef Environment

**DOI:** 10.3389/fmicb.2019.01860

**Published:** 2019-08-14

**Authors:** Chloé A. Pupier, Vanessa N. Bednarz, Renaud Grover, Maoz Fine, Jean-François Maguer, Christine Ferrier-Pagès

**Affiliations:** ^1^Marine Department, Centre Scientifique de Monaco, Monaco, Monaco; ^2^Collège Doctoral, Sorbonne Université, Paris, France; ^3^The Mina and Everard Goodman Faculty of Life Sciences, Bar-Ilan University, Ramat Gan, Israel; ^4^The Interuniversity Institute for Marine Science in Eilat, Eilat, Israel; ^5^Laboratoire de l’Environnement Marin (LEMAR), UMR 6539, UBO/CNRS/IRD/IFREMER, Institut Universitaire Européen de la Mer, Plouzané, France

**Keywords:** dinitrogen fixation, diazotrophs, scleractinian corals, soft corals, Red Sea

## Abstract

Corals are associated with dinitrogen (N_2_)-fixing bacteria that potentially represent an additional nitrogen (N) source for the coral holobiont in oligotrophic reef environments. Nevertheless, the few studies investigating the assimilation of diazotrophically derived nitrogen (DDN) by tropical corals are limited to a single scleractinian species (i.e., *Stylophora pistillata*). The present study quantified DDN assimilation rates in four scleractinian and three soft coral species from the shallow waters of the oligotrophic Northern Red Sea using the ^15^N_2_ tracer technique. All scleractinian species significantly stimulated N_2_ fixation in the coral-surrounding seawater (and mucus) and assimilated DDN into their tissue. Interestingly, N_2_ fixation was not detected in the tissue and surrounding seawater of soft corals, despite the fact that soft corals were able to take up DDN from a culture of free-living diazotrophs. Soft coral mucus likely represents an unfavorable habitat for the colonization and activity of diazotrophs as it contains a low amount of particulate organic matter, with a relatively high N content, compared to the mucus of scleractinian corals. In addition, it is known to present antimicrobial properties. Overall, this study suggests that DDN assimilation into coral tissues depends on the presence of active diazotrophs in the coral’s mucus layer and/or surrounding seawater. Since N is often a limiting nutrient for primary productivity in oligotrophic reef waters, the divergent capacity of scleractinian and soft corals to promote N_2_ fixation may have implications for N availability and reef biogeochemistry in scleractinian versus soft coral-dominated reefs.

## Introduction

Coral reefs are highly productive ecosystems despite thriving in oligotrophic waters, which contain low levels of essential dissolved inorganic nutrients such as nitrogen (N) (de Goeij et al., 2013). Corals, which are the main reef builders, can achieve high productivity thanks to their association with endosymbiotic dinoflagellates, which recycle the animal waste products and are efficient in scavenging inorganic N dissolved in seawater (Wang and Douglas, 1999; Grover et al., 2003). Corals also profit from high rates of N recycling in the water column and sediments *via* microbial processes (Carpenter and Capone, 2008), and from biological dinitrogen (N_2_) fixation of diazotrophic symbionts (Bednarz et al., 2017), which are heterotrophic bacteria and cyanobacteria that convert atmospheric N_2_ into bioavailable N. In addition to being associated with benthic organisms (reviewed in Benavides et al., 2017), diazotrophs can live pelagic, and colonize reef substrates (e.g., sediments, coral rubble). Many studies using the acetylene reduction assay (ARA) method have recorded high rates of N_2_ fixation either in reef waters or in the presence of corals (i.e., Bednarz et al., 2015; Rädecker et al., 2015; Cardini et al., 2016). The ARA technique quantifies gross N_2_ fixation without providing insights into DDN (diazotrophically derived nitrogen) assimilation by the coral-dinoflagellate symbiosis. Instead, the use of labeled ^15^N_2_ gas measures DDN assimilation (net N_2_ fixation) and distinguishes between the different compartments (i.e., seawater particles, tissue, dinoflagellate symbionts), but it is still poorly known to what extent and under which conditions corals profit from DDN.

Most of the research using the ^15^N_2_ method to assess DDN assimilation in adult tropical corals has been directed toward only one species, *Stylophora pistillata*, belonging to the Pocilloporidae family. These studies showed that *S. pistillata* can acquire DDN *via* grazing on a culture of diazotrophic cells (Benavides et al., 2016). However, under natural conditions, this coral species has variable capacities to stimulate diazotrophic activity in seawater as well as variable DDN assimilation rates into the coral holobiont (Grover et al., 2014; Bednarz et al., 2017; Lesser et al., 2018). As DDN can play a major role in supplying N to corals (Cardini et al., 2016; Bednarz et al., 2017), the estimation of the capacity of different coral species to take advantage of this nutrient source can help in understanding how they cope with nutrient limitation. In addition, no study so far investigated DDN assimilation by soft corals, although they represent together with scleractinian corals the two most dominant benthic groups on many reefs worldwide. Soft corals can quickly colonize open space due to their fast growth rates, high fecundity, and asexual reproduction and these opportunistic life history features may be facilitated by diazotrophs as additional N source (i.e., DDN). The aim of this study was to perform a multi-species comparison and to investigate the capacity of different scleractinian and soft coral species to either assimilate DDN/diazotrophic cells and/or transfer it to the surrounding seawater.

## Materials and Methods

### Laboratory Experiment: Diazotrophically Derived Nitrogen Assimilation From External Diazotrophs

To assess whether scleractinian and soft corals have the same capacities to assimilate DDN from external active diazotrophs in the seawater, an experiment was performed at the Monaco Scientific Centre. We used the scleractinian coral *S. pistillata* and the soft coral *Sarcophyton* sp., which have been maintained in aquaria for years, and are not associated with active diazotrophs (Bednarz, unpublished data; Kooperman et al., 2007). All colonies were maintained under the same light (200 μmoles photons m^−2^ s^−1^) and temperature (25°C) conditions to ensure comparability between the species during the following experiment. For this purpose, a culture of diazotrophs (*Crocosphaera watsonii*) was prepared and diluted to obtain two cell concentrations of 100 and 1,000 cells ml^−1^, respectively, matching concentrations of 3- to 20-μm size class phycoerythrin-containing unicellular cyanobacteria under non-bloom and bloom conditions (Campbell et al., 1997). *C. watsonii* concentrations were determined using a hemocytometer (Z1 particles counter, Beckman Coulter, USA). Four nubbins of each coral species per *C. watsonii* concentration were then individually placed in 320-ml gas-tight bottles completely filled with 280 ml of 0.22 μm-filtered seawater amended with the adequate concentration of diazotrophs and 40 ml of ^15^N_2_ enriched seawater. In addition, three nubbins of each species were incubated under the same above conditions but without *C. watsonii* cells. At the end of the 24 h incubation, corals were removed and stored frozen until subsequent analysis. They were then processed as described below.

### Field Experiment: Diazotrophically Derived Nitrogen/Diazotrophic Cells Assimilation and Transfer to Seawater

#### Biological Material

The study was conducted during November 2017 at the Interuniversity Institute (IUI) of Marine Sciences, Eilat, Red Sea. Coral fragments were collected by SCUBA diving from different colonies of the reef adjacent to the IUI and were brought back to the Red Sea Simulator facility (see Bellworthy and Fine, 2018, for more details regarding the system) to recover for one day prior to starting the incubations. Scleractinian corals (*Acropora eurystoma*, *n* = 6; *Pocillopora damicornis*, *n* = 3; *Goniastrea sp.*, *n* = 3; and *Cynarina sp.*, *n* = 2) were sampled in shallow waters (8–10 m depth) and soft corals (*n* = 5 for all species; *Dendronephthya* sp., *Rhytisma fulvum fulvum* and *Litophyton* sp.) in shallow waters and at upper mesophotic depths (40–45 m). Biological replicates for each species were derived from individual colonies and the different species were chosen in order to cover a broad range of families and morphological traits (Table 1). Three additional nubbins per species per depth were collected to measure natural ^15^N abundance of the corals. At the collection time, the site was characterized by stable temperature (24–25°C) and nutrient levels (< 0.5 μM dissolved N and < 0.2 μM dissolved phosphorus) along the depth gradient (data from the Israel National Monitoring program of the Gulf of Eilat, http://www.iui-eilat.ac.il/Research/NMPMeteoData.aspx).

**Table 1:** Scleractinian and soft coral species investigated in the study.

Coral group	Species	Family	Morphology
Scleractinian	*Acropora eurystoma*	Acroporidae	Branching
	*Cynarina* sp.	Lobophyliidae	Mounding
	*Goniastrea* sp.	Merulinidae	Mounding
	*Pocillopora damicornis*	Pocilloporidae	Branching
Soft	*Dendronephthya* sp.	Nephtheidae	Arborescent
	*Litophyton* sp.	Nephtheidae	Arborescent
	*Rhytisma fulvum fulvum*	Alcyoniidae	Mat-forming

#### N_2_ Fixation Measurements

The ^15^N_2_ seawater addition method was used to assess N_2_ fixation rates. For this purpose, ^15^N_2_-enriched seawater was produced prior to the incubation experiment by injection of 10 ml of ^15^N_2_ gas (98% Eurisotop) into gas-tight 250-ml bottles completely filled with degassed, 0.2 μm-filtered seawater, followed by vigorous shaking for 12 h to ensure 100% ^15^N_2_ equilibration (Mohr et al., 2010). In order to test which coral species can assimilate DDN or can enrich the seawater in DDN, collected nubbins from each coral species were individually placed in gas-tight bottles of volume 600 ml. Bottles were completely filled with 120 μm-filtered seawater, directly pumped from the reef, with 10% replaced by ^15^N_2_-enriched seawater (resulting in theoretical enrichment of ~9.8 atom%). A first set of three control bottles was prepared as described above but without corals to measure the baseline of N_2_ fixation by planktonic diazotrophs. To evaluate the natural ^15^N abundance of the corals, a second set of controls consisted in incubating three coral nubbins from each species (only two for *Cynarina* sp.) in 120 μm-filtered seawater incubations without ^15^N_2_ addition. All bottles were incubated for 24 h in several outdoor aquaria. The seawater temperature was kept constant at *in situ* temperature (~ 24°C) by using continuous supply of seawater in the aquaria. Corals were exposed to the natural diel light cycle and shaded to the corresponding irradiance of their collection site by applying layers of black mesh above the aquaria and considering an attenuation coefficient equal to 0.072–0.1 m^−1^ (Akkaynak et al., 2017; Tamir et al., 2019). At the end of the incubations, corals were removed from seawater and stored frozen until subsequent analysis. Incubation water of all bottles was filtered onto pre-combusted (450°C for 5 h) GF/F filters which were dried at 60°C for 48 h. Corals and filters were processed as described in the sample analysis section.

### Sample Analysis

For scleractinian corals, the tissue was removed from the skeleton using an air brush and homogenized with a potter tissue grinder. The host tissue and zooxanthellae were separated through a series of centrifugations according to Grover et al. (2003) and each fraction was freeze-dried. Soft corals were treated as described in Pupier et al. (2018). Briefly, they were freeze-dried, the resulting powder homogenized in distilled water, and then separated in several centrifugation steps into the host tissue and zooxanthellae fractions as described above. Each fraction was subsequently freeze-dried again. The ^15^N enrichment as well as particulate organic carbon (POC) and particulate N content (PN) of each sample and filter were determined using a mass spectrometer (Delta Plus; Thermo Fisher Scientific, Germany) coupled to a C/N analyzer (Flash EA; Thermo Fisher Scientific, Germany). To calculate N_2_ fixation in particles of the incubation water or DDN assimilation by corals (host and/or zooxanthellae), the equation of Montoya et al. (1996) was used:

$$N_{2}\;\mathrm{fixation}\;\mathrm{or}\;DDN\;\mathrm{assimilation}=\frac{\mathrm{atom}{\%}^{15}N_{\mathrm{excess}}\times {PN}_{\mathrm{sample}}}{t\times 9.8}$$

where *t* is the incubation time, 9.8 the initial ^15^N enrichment of the incubation water, and PN_sample_ the particulate N content of the samples. For each sample, the atom% ^15^N_excess_ enrichment was calculated by subtracting the natural ^15^N enrichment of control samples without ^15^N_2_ exposure (atom% ^15^N_control_) from the ^15^N enrichment of samples after exposure to ^15^N_2_-enriched seawater (atom% ^15^N_sample_). The atom% ^15^N_sample_ was considered significant when it was at least three fold higher than the standard deviation of the atom% ^15^N_control_. N_2_ fixation and DDN assimilation values were normalized to the volume of water filtered or to the total dry weight of the sample. For comparison with previous studies, scleractinian corals data were also normalized to the skeletal surface area determined using the wax technique (Veal et al., 2010).

### Statistical Analyses

Analyses were performed using R software (R Foundation for Statistical Computing). All data were expressed as mean ± standard error. Prior to analyses, outlier values were identified using Grubb’s test and were excluded when *p*’s were significant (*p* < 0.05). Assumptions of normality and homoscedasticity of variance were evaluated through Shapiro’s and Bartlett’s tests. A non-parametric Kruskal-Wallis test was used to test for differences between groups (hard vs. soft corals) and depths (shallow vs. deep soft corals) on POC and POC:PN. Analyses of variance (ANOVAs) were performed to respectively test the effect of species and morphologies on DDN transfer and assimilation. Tukey tests were performed as *a posteriori* testing.

## Results

**Figure 1:**
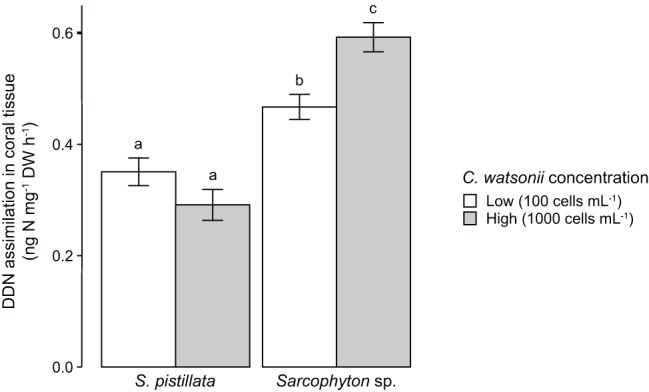
Scleractinian and soft corals’ DDN assimilation in the presence of two different concentrations of active diazotrophs (*Crocosphaera watsonii*) in seawater. Investigated species are *Stylophora pistillata* (scleractinian coral) and *Sarcophyton* sp. (soft coral). Error bars indicate standard error. Letters refer to Tukey’ HSD testing. DDN, diazotrophically derived nitrogen; DW, dry weight.

In the laboratory experiment, no N_2_ fixation (neither in seawater nor in coral tissue) was measured when corals were incubated without the addition of *C. watsonii* cells to the seawater. Both *S. pistillata* and *Sarcophyton* sp. however demonstrated abilities to assimilate DDN in the presence of active diazotrophs (i.e., *C. watsonii*) in seawater (Figure 1). Overall, *Sarcophyton* sp. assimilated 65% more DDN in its tissue than *S. pistillata*. While there was no difference between the DDN assimilations of *S. pistillata* exposed at the two cell concentrations (Tukey HSD: *p* = 0.389), *Sarcophyton* sp. significantly increased its DDN assimilation under higher cell concentration (Tukey HSD: *p* = 0.020).

**Figure 2:**
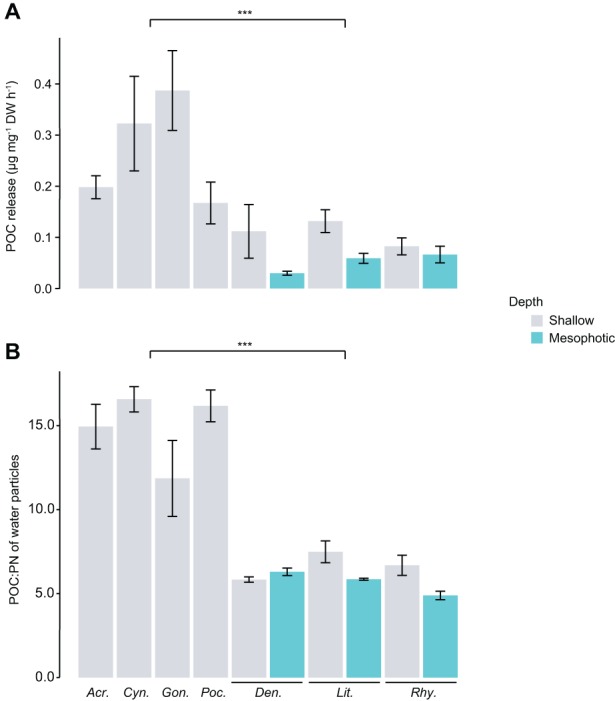
POC release rates by corals **(A)** and POC:PN ratio of released particles **(B)**. POC, particulate organic carbon; PN, particulate nitrogen. DW, dry weight. Asterisks indicate a significant difference between the two groups (scleractinian vs. soft corals, *p* < 0.001). From left to right: *Acr*., *Acropora cervicornis*; *Cyn*., *Cynarina* sp.; *Gon*., *Goniastrea* sp.; *Poc*., *Pocillopora damicornis*; *Den*., *Dendronephthya* sp.; *Lit*., *Litophyton* sp.; *Rhy*., *Rhytisma fulvum fulvum*.

**Figure 3:**
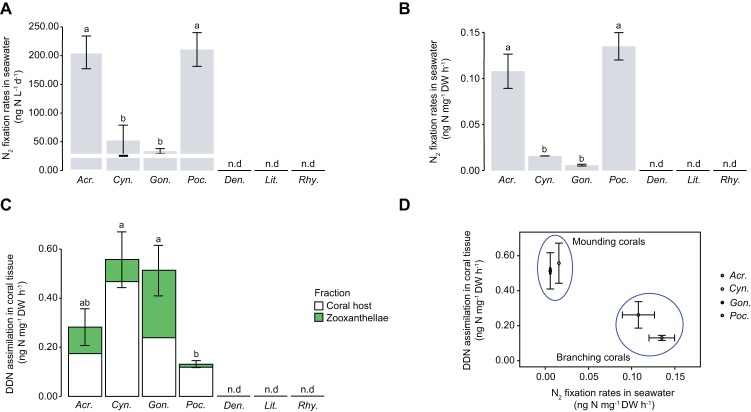
N_2_ fixation in seawater **(A, B)**, DDN assimilation in coral tissue **(C)**, and relationship between the two **(D)**. The white line indicates N_2_ fixation rate occurring in 120 μm-filtered seawater incubated without corals. Error bars indicate standard error. n.d., not detected; DDN, diazotrophically derived nitrogen; DW, dry weight. From left to right: *Acr*., *Acropora cervicornis*; *Cyn*., *Cynarina* sp.; *Gon*., *Goniastrea* sp.; *Poc*., *Pocillopora damicornis*; *Den*., *Dendronephthya* sp.; *Lit*., *Litophyton* sp.; *Rhy*., *Rhytisma fulvum fulvum*.

In the field experiment, a very low level of N_2_ fixation was measured in seawater with natural populations of diazotrophs, in the absence of corals (Figure 2A). All coral species enriched the seawater in mucus-containing particles during the 24-h incubation (Figure 2A). POC, which is a proxy for living and detrital particles present in the water and being released by the corals, was two- to seven-fold higher in chambers containing scleractinian corals compared to those containing soft corals (Kruskal-Wallis: *p* < 0.001). There was no difference in POC release between shallow and mesophotic soft corals (Kruskal-Wallis: *p* = 0.138). Particles released by scleractinian corals presented a higher POC:PN ratio (between 11.9 and 16.6) compared to those of soft corals (between 4.9 and 7.5) (Figure 2B, Kruskal-Wallis: *p* < 0.001). While there was no N_2_ fixation in the incubation water containing all soft coral species sampled in shallow or mesophotic environments, a significant fixation was observed in the incubation water of all four scleractinian species (Figures 3A,B). Fixation rates ranged from 34 to 211 ng N L^−1^ over the 24-h incubation, or between 6 and 135 10^−3^ ng N mg DW^−1^ h^−1^ when expressed per coral biomass. For both normalizations, rates were significantly different between species (ANOVA: *p* = 0.003 and *p* = 0.002, respectively) and morphologies (branching vs. mounding, ANOVA: *p* < 0.001) with higher rates obtained for *A. eurystoma* and *P. damicornis*. Significant assimilation of DDN was observed in the host tissue and zooxanthellae of scleractinian corals, but not for the soft coral species from either shallow or mesophotic environments (Figure 3C). The DDN assimilation in host tissue was equal or higher than the assimilation in the zooxanthellae fraction. Moreover, an inverse trend was observed between N_2_ fixation occurring in incubation water and DDN assimilated by the whole symbiotic association (Figure 3D).

## Discussion

### Divergent Capacity of Coral Mucus to Enrich Seawater With Diazotrophically Derived Nitrogen

This study first highlights contrasting capacities of scleractinian and soft corals to promote N_2_ fixation in seawater. Freshly fixed N_2_ was traced in the incubation water containing scleractinian coral species, while no fixation in the seawater occurred in presence of soft corals. A previous study, which has used the ARA method, has also recorded much lower rates of gross N_2_ fixation in the presence of soft compared to scleractinian corals (Bednarz et al., 2015). A difference in the quality and/or quantity of mucus (particulate and dissolved organic carbon, POC and DOC) released by the two coral groups as observed here together with a different amount of mucus-associated bacteria most likely explain differences in seawater diazotroph abundance or activity. While the sugar composition of the mucus can be similar between soft and scleractinian corals (Meikle et al., 1988; Hadaidi et al., 2019), both the amount and POC:PN ratio of mucus are often species-dependent (Naumann et al., 2010; Hadaidi et al., 2019). Pogoreutz et al. (2017) highlighted that DOC enrichment, in the form of sugars, can significantly stimulate diazotrophic N_2_ fixation. In our study, scleractinian corals released between 1.9 and 8.9 mg POC m^−2^ h^−1^, which is in the range of what has been reported for Red Sea scleractinian corals (between 0.3 and 6.5 mg POC m^−2^ h^−1^) by Naumann et al. (2010). Soft corals however released POC at two- to ten-fold lower rates. This is in agreement with previous measurements performed on another Red Sea soft coral belonging to the Xeniidae family, for which no POC release was observed (Bednarz et al., 2012). As POC is a proxy for detrital and living particles, the lower particle content in the soft coral surrounding seawater can be explained by the well-known antimicrobial properties of soft coral mucus (Kelman et al., 2006; Nakajima et al., 2018). These authors indeed demonstrated that little antimicrobial activity was measured for scleractinian coral mucus whereas soft corals, including those studied in this work, significantly inhibited the growth of co-occurring seawater bacteria through the production of antibiotic compounds. Hence, scleractinian corals release mucus with a large number of bacteria into the surrounding seawater that can influence the activity and diversity of planktonic diazotroph populations. This feature may be greatly reduced in soft corals, as their high antimicrobial activity constitutes a defense strategy against invading pathogens and fouling organisms for the holobiont, thus possibly assisting in competition over space and nutrition (Kelman et al., 2009). Finally, the organic matter (OM) released by soft corals presented a particularly low POC:PN ratio (of 5–7, i.e., particles enriched in N) compared to the OM released by scleractinian corals (from 12–17 in this study, range in agreement with Naumann et al., 2010). This is also in agreement with the findings of two previous studies (Meikle et al., 1988; Nakajima et al., 2018), which observed a higher protein and lower carbohydrate composition of soft coral mucus compared to scleractinian coral mucus. Since N_2_ fixation is rather promoted by N deprivation (Knapp, 2012), a low POC:PN ratio is not in favor of N_2_ fixation. Put together, our results suggest that the lack of N_2_ fixation in seawater surrounding soft corals is potentially due to the antimicrobial properties of soft coral mucus and the N repletion of the released particles. On the contrary to soft corals, seawater N_2_ fixation was recorded in presence of scleractinian corals. Measured rates (0.01–0.13 ng N mg^−1^ DW h^−1^ or 0.03 nmol N cm^−2^ h^−1^ or 2 to 15 nmol N L^−1^ d^−1^) are however significantly lower than those recorded for other benthic substrates such as sediment, sands, or microbial mats (reviewed in Benavides et al., 2017). Nevertheless, they are in the same range as those previously measured with seawater diazotrophs of the Great Barrier Reef (from 5 to 70 nmol N L^−1^ d^−1^, Messer et al., 2017).

### Divergent Capacity of Corals to Assimilate Diazotrophically Derived Nitrogen

Our results demonstrate that all investigated scleractinian species assimilated DDN, since ^15^N enrichment was observed in both host tissue and zooxanthellae. In contrast, no ^15^N enrichment of soft coral tissue was detected either in corals collected from shallow or mesophotic reefs. The fact that even mesophotic soft coral colonies did not assimilate DDN stands in contrast to the scleractinian species *S. pistillata*, which shows higher assimilation rates in deep compared to shallow waters (Bednarz et al., 2017). Here, DDN assimilation rates of scleractinian corals (ca. 0.3– ng cm^−2^ h^−1^ or 0.6–1.7 nmol cm^−2^ d^−1^) are in agreement with those measured for the species *S. pistillata* sampled in shallow waters of the Red Sea or the Great Barrier Reef (Bednarz et al., 2017; Lesser et al., 2018). However, they were 6–10 times lower than those measured for corals depending more on heterotrophy, such as bleached corals or those living in mesophotic and temperate environments (Bednarz et al., 2017, 2019). These observations suggest that the contribution of DDN to the N requirements of corals increases during nutrient deprivation, or when the uptake of other inorganic N forms by dinoflagellate symbionts is reduced (Bednarz et al., 2019). The inverse trend observed in this study between seawater N_2_ fixation and DDN assimilation rates in corals suggests that a substantial part of the DDN assimilated by corals is obtained from heterotrophic feeding on fixed N compounds and/or from diazotrophic cells growing in the mucus layer (Bednarz et al., 2017). Therefore, the lack of N_2_ fixation in the surrounding seawater of soft corals (highlighting the absence of active diazotrophs in their mucus) may also explain why we did not detect any DDN assimilation by shallow and mesophotic soft corals. Furthermore, coral polyps with a mounding morphology show generally higher heterotrophic feedings rates as compared to those with a branching morphology (Palardy et al., 2005). This corresponds to the higher DDN assimilation rates observed for *Cynarina* sp. and *Goniastrea* sp. (mounding morphology) as compared to *A. cervicornis* and *P. damicornis* (branching morphology). Corals can receive DDN not only from mucus-associated but also from pelagic diazotrophs (Camps et al., 2016). In our experiment on *C. watsonii* cells, both corals assimilated DDN (i.e., in the form of N_2_-fixing *C. watsonii* cells or DDN compounds released by *C. watsonii* cells) from the surrounding seawater with rates similar to those observed for our corals from the Red Sea. Moreover, *Sarcophyton* sp. assimilated more DDN in the high versus the low *C. watsonii* concentration suggesting that the assimilation capacity of this species was not saturated even under simulated bloom conditions. This indeed indicates that soft corals also have the capacity to assimilate diazotrophs/DDN from the seawater. Thus, the lack in DDN assimilation by Red Sea soft corals sampled in the field, including the Symbiodiniaceae-free and heterotrophic genus *Dendronephthya* sp., is likely not due to a lower heterotrophy as compared to scleractinian corals, but rather linked to the absence of active diazotrophs in the soft coral mucus. For example, strains of putative diazotrophs (*Vibrio campbellii* and *Vibrio parahaemolyticus*; Chimetto et al., 2008) have been isolated from *Dendronephthya* sp. (Harder et al., 2003). We therefore hypothesize that even if diazotrophs can be present, the mucus of soft corals does not represent a favorable habitat for diazotrophic activity. Moreover, non-negligible DDN assimilation rates have been detected in the skeleton of scleractinian corals due to the activity of endolithic diazotrophs (Sangsawang et al., 2017; Bednarz et al., 2019). Such contribution to the total N budget of the holobiont cannot be observed in soft corals as they lack a calcium carbonate skeleton. *In situ* experiments remain to be investigated to assess whether soft corals benefit from any other external source of N on reefs that could enhance their opportunistic life history features.

## Conclusion

It is widely accepted that N is one of the most limiting nutrients for reef primary productivity (Eyre et al., 2008), and that benthic N_2_ fixation plays an important role in supplying bioavailable N within benthic and pelagic reef habitats (Cardini et al., 2016; Messer et al., 2017). The surface structure of benthic organisms and substrates provides an important habitat for the colonization by diazotrophs, but the abundance, composition, and activity of the diazotrophic community may depend on the type of organism/substrate. Here, we suggest that soft coral mucus represents likely a less favorable habitat for microbes as compared to scleractinian coral mucus due to its relatively low C but high N content along with antimicrobial properties. The resulting different capacity of scleractinian and soft corals to promote active diazotroph populations and N_2_ fixation in reef waters may have several implications for N availability and reef biogeochemistry in the future. Particularly, coral reefs that have experienced phase shifts from hard to soft coral dominance (Stobart et al., 2005; Norström et al., 2009; Pratchett, 2010; Inoue et al., 2013) may suffer from a significant decrease in N_2_ fixation and subsequent N limitation. This may ultimately affect primary production, carbon sequestration, and the functioning of coral reef ecosystems and could be an interesting topic for future studies.

## Data Availability

All datasets generated for this study are included in the manuscript and/or the supplementary files.

## Author Contributions

CF-P, CP, RG, and VB conceived the experiment. J-FM and RG performed the mass spectrometry analyses. All authors analyzed the data and wrote the manuscript. CF-P, J-FM, and MF acquired funding.

### Conflict of Interest Statement

The authors declare that the research was conducted in the absence of any commercial or financial relationships that could be construed as a potential conflict of interest.
